# Data on host specificity and symbiotic association between indigenous *Rhizobium* BD1 strain and *Vigna radiata* (green gram)

**DOI:** 10.1016/j.dib.2021.107520

**Published:** 2021-10-27

**Authors:** Sanjeev Kumar K, Pavan Kumar Pindi

**Affiliations:** Department of Microbiology, Palamuru University, Mahabubnagar, Telangana, India. 509 001

**Keywords:** Green gram, Host specificity, Inoculants, Nodulation, *Rhizobium* sp. BD1 strain, *Vigna radiata*

## Abstract

The main objective of this study is to use bio-inoculants in relative to specific legume plant diversity for, enhanced nodulation and plant growth. Method involves organically based selection of 36 rhizobial strains, of which 6 strains were isolated to assess the efficiency of relative host-specific inoculation on nodulation and development in legumes viz. *Vigna radiata.* All promising combinations of the preferred rhizobial strain inoculants were tested under sterile conditions for improving nodulation and to screen the best isolate to be evaluated for its enhanced characteristics through inoculation by field trial in various soils. It was observed that the strains from Bhadrachalam forest BD1 are highly host specific for *Vigna radiata* plants and when inoculated, improved nodulation and enhanced plant growth. Because of the novel characters in BD1, further studies were carried out and was identified as *Rhizobium* sp. BD1 (NCBI Accession no. MT577595). The percentage of nitrogen content in *Vigna radiata* ranged between 1.2% to 2.9%. This *Rhizobium* sp. BD1 was tested for the unraveling and amelioration of crop production in barren, polluted and agricultural soils which showed enhanced characteristics in *Vigna radiata* plants. This method may be employed across the globe of same climatic conditions for the retrieval of plants in soils that carry agriculture unsuccessfully.

## Specifications Table

**Table:** 

Subject	Microbiology
Specific subject area	Agricultural microbiology: Bioinoculants
Type of data	Table Graph Figure
How data were acquired	Experimental Data: Nitrogen estimation [4], Phosphorous estimation [3].
Data format	Analytical data
Parameters for data collection	Fresh and dry weight; Length of Root and shoot; Nodules: Number, size, weight; Nitrogen, Phosphorous, content recorded after 60 days after sowing.
Description of data collection	Experimental observation: *Vigna radiata* plants were grown in various forest soil samples; the best grown plants were selected for isolation of the *Rhizobium* sps. from the nodules. Parameters like weight, length, protein and Nitrogen content were recorded and analysed.
Data source location	Palamuru University Mahabubnagar, Telangana India
Data accessibility	Data include in this article

## Value of the Data

• The data explains that host specificity and strain efficiency do exist between Microbial-plant interactions.
• Scope for further studies on efficiency of bioinoculants.
• Helps in understanding: appropriate bioinoculant is essential to get better yield.
• This data encourages the farmers to employ bioinoulants and a scope for small scale industries who are interested in establishing bioinoculants as source of income.
• This brings into lime light why still bioinoculants are a supplement to chemical fertilizers but not a replacement.
• This leading a scope to develop and design an efficient bioinoculants, an eco-friendly product, for various crops. Thus, replacing or supplementing the chemical fertilizers, and sustainable development in agriculture.

## 1 Data Description

.

**Graph 1 and 2:**
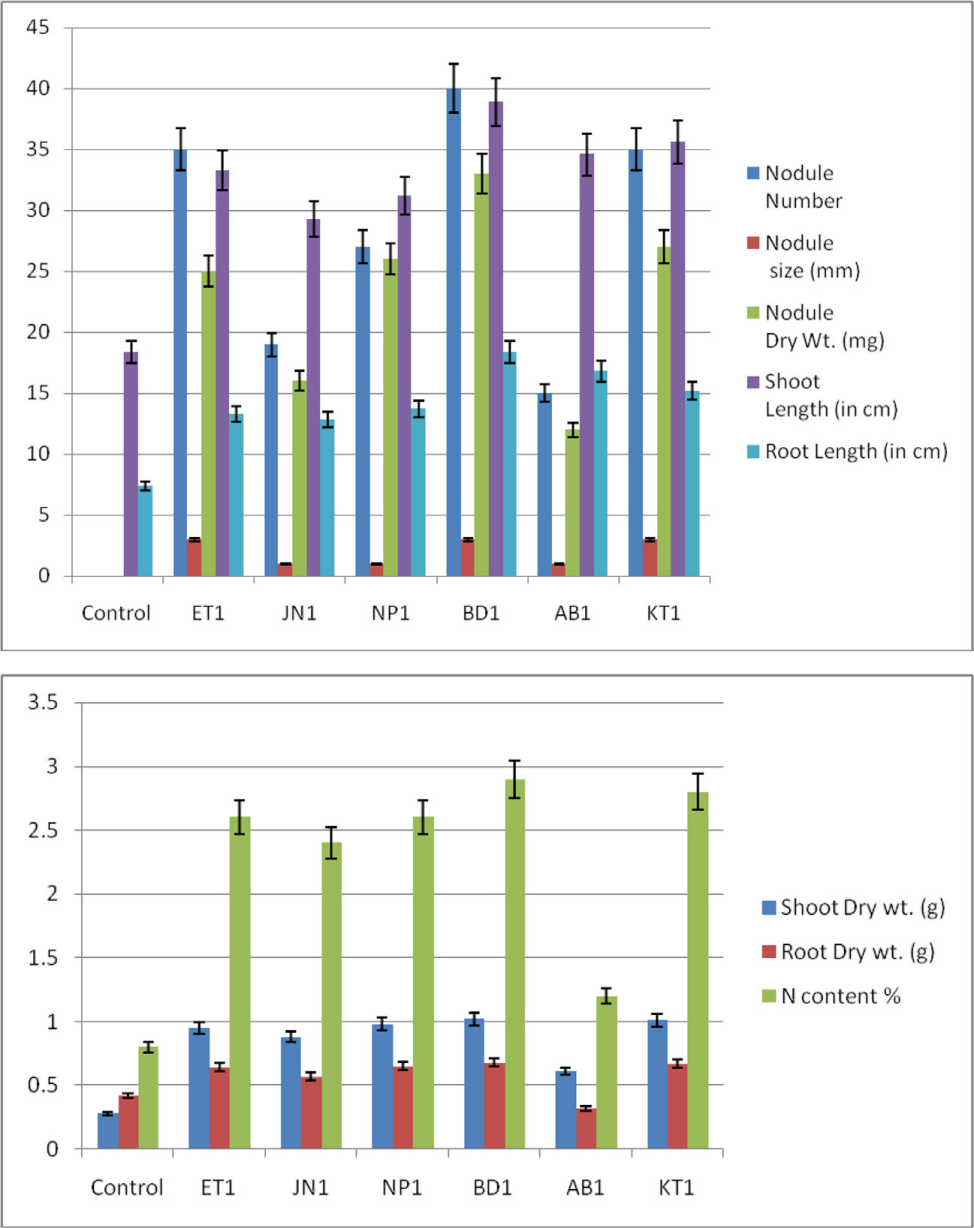
Represents the parameters of nodule (Number, Size and dry weight) developed on *Vigna radiata* plant and height of the plant with various rhizobial strains.

**Graph 3:**
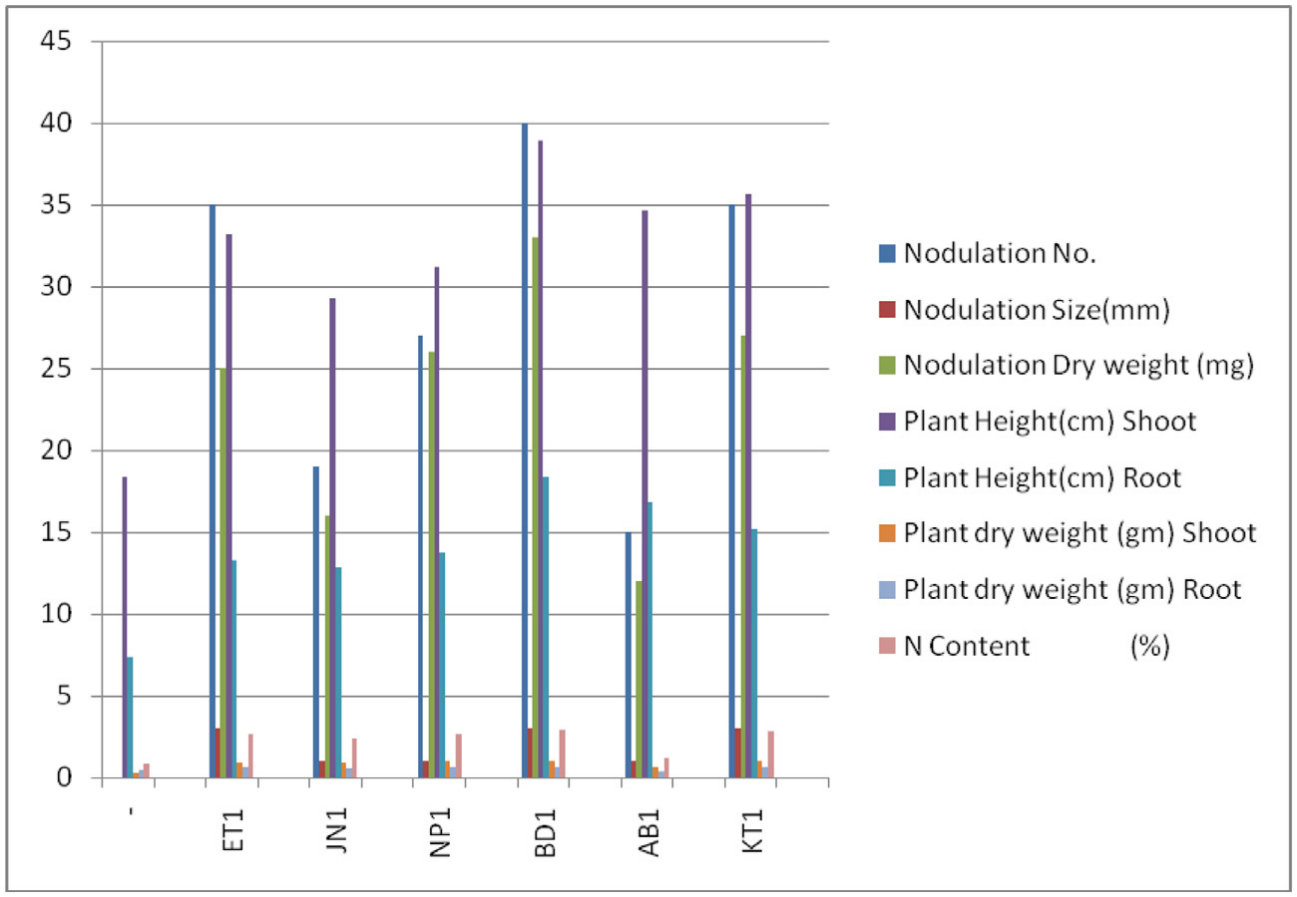
Represents the parameter of *Vigna radiata* upon inoculation with different rhizobial strains.

**Graph 4:**
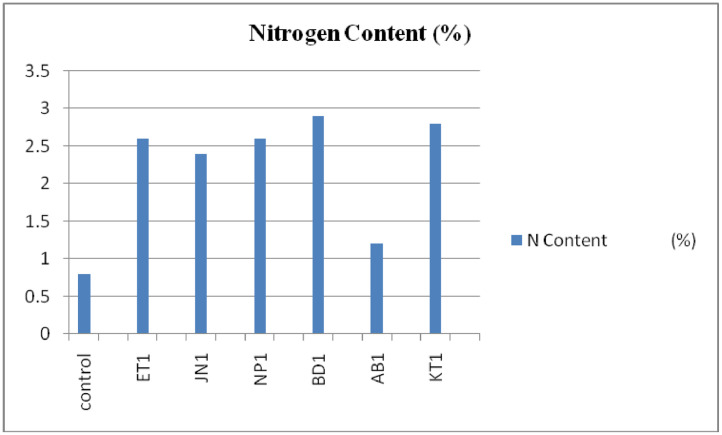
Represents the Variation in percentage of Nitrogen accumulation with various strain inoculations (Treatments).

**Graph 5:**
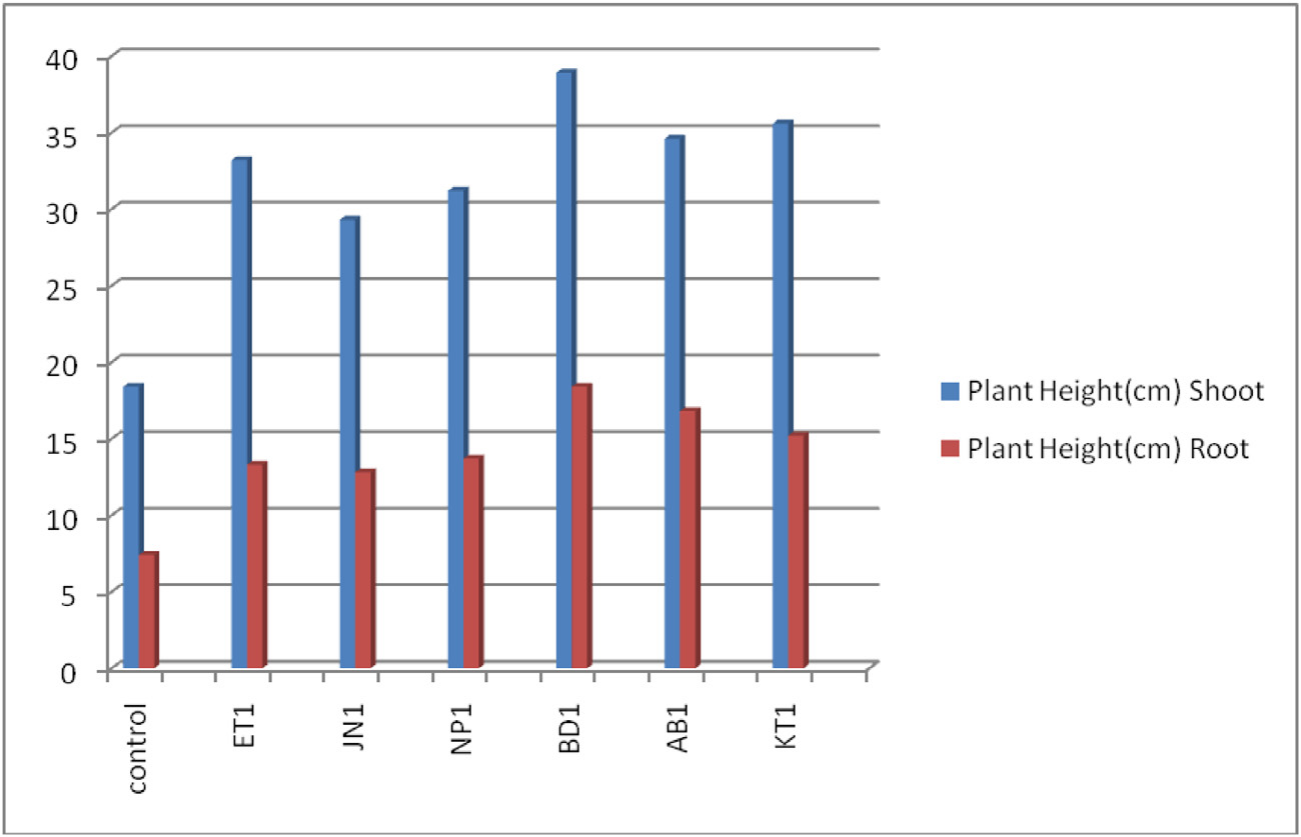
Represents the effect of rhizobium strains on *Vigna radiata* plant height of shoot and root.

## 2 Experimental Design, Materials and Methods

### 2.1 Materials and methods

#### 2.1.1 Selection of rhizosphere soils

Undisturbed rhizosphere soils along with relative six legume species [*Arachis hypogaea* (Ground nut), *Glycine* max (Soya bean), *Cicer arietinum* (Chickpea), *Phaseolus vulgaris* (Common bean), *Vigna radiata* (Green gram), *Cajanus cajan* (pigeon pea)] in triplets were collected from six forest soils of Jannaram (JN)- Adilabad, Eturunagaram (ET)-Warangal, Narsapur (NP)-Medak, Bhadrachalam(BD)-Khammam, Amrabad (AB)-Mahabubnagar and Kataram (KT)-Karimnagar areas.

#### 2.1.2 Physico-chemical analysis of soil

These rhizosphere soils were not having any previous history of chemical fertilizers, so there was no chance of growth inhibition of natural bioinoculants by the action of chemical fertilizers. Soil available nitrogen was estimated by alkaline potassium permanganate method [2], and available phosphorous was determined after [1]. Potassium determined by flame photometrically [3]. Obtained results were tabulated in Table 1.

**Table 1:** Physico-chemical characteristics in 6 forest soils (NPK in kg/hectare).

	Coordinates						
Sample collected from	Latitude	Longitude	Soil type	N	P	K	pH
Jannaram	19.1156 N	78.999 E	Red soil	265.06	105.19	160.65	6.9
Eturnagaram	18.338478	80.42698	Red soil	223.01	110.21	150.05	6.3
Narsapur	17.738	78.2845	Deep black soil	272.73	112.32	160.00	6.2
Bhadrachalam	17.66879	80.89359	Red soil clay	273.03	118.11	157.02	6.7
Kataram	17.54458	80.64689	Red soil	272.08	104.03	147.00	6.2
Amrabad	16.383	78.833	Sandy loam	244.70	27.20	451.55	8.3

#### 2.1.3 Isolation of rhizobia from nodules

A healthy plant was uprooted with intact soil around the roots. Roots were then carefully washed with a jet of water. Nodules which are pink multilobed and situated on the top of the root were selected for isolation of *Rhizobium*. Nodule is separated from the root carefully so that piece of root on the side of the nodule remain attached and the nodule is not injured. The nodules were thoroughly washed with running tap water placing it a tube with a nylon mesh on one end. The other end of the tube was connected to tap for about five minutes.

Thoroughly washed nodules were transferred to a sterile test tube and treated with 0.1% HgCl_2_ and 70% ethyl alcohol, for 3 min and one min respectively. The test tube is shaken periodically in order to remove the adhering air bubbles and the fresh sterilant gets in touch with the nodules. After three minutes, HgCl_2_ solution is decanted off and nodule was immersed in alcohol for 1 min. After the nodule surface gets sterilized it was washed with sterile water for at least ten times so as to remove the sterilants completely. Nodules were crushed with a sterile glass rod with flat end. Care was taken so that test tube may not break during crushing. Suspension obtained after crushing of nodule was used for isolation of *Rhizobium*. Serial dilution of the same was done and 10^−6^ dilution was selected for isolation.

Yeast extract mannitol agar (YEMA) was used as a selective medium for isolation of *Rhizobium* sp. 100 µl of 10^−6^ dilution was inoculated on sterile YEMA media plates by spread plate technique and incubated at 28 °C overnight. Based on the color of the colony and other characteristics, *Rhizobium* was isolated and various other confirmatory tests were performed.

### 2.2 Confirmatory tests

#### 2.2.1 Congo red test

Congo red can aid the identification of rhizobia from other kinds of bacteria. In broad the Rhizobia absorb the stain weakly while several of the common soil bacteria take it up strongly.

#### 2.2.2 Growth on alkaline medium

*A. radiobacter* can be isolated by streaking on Hoffer's alkaline medium (pH 11) in which *Rhizobium* growth is inhibited, while *A. radiobacter* grow. YEMA added with 1 ml/lit of thymol blue (1.6% sol.) is adjusted to pH 11. On slants, the growth of *A. radiobacter* and the change in color of indicator is observed upto 15 days. If no growth (or) change in color is observed, it may be *Rhizobium*.

#### 2.2.3 Growth in glucose peptone agar

**Glucose peptone agar** (Glucose 10 g, Peptone 20.0 g, NaCl 5.0 g, Agar - 15.0 g, Bromo cresol purple 1.0 ml (1.6% alcoholic sol.) pH 7.1) was used to distinguish rhizobia, which generally show little growth on the medium without changing the pH, whereas *Agrobacteria* grow well shows enormous growth. Observations were taken after 15 days of incubation for growth and change in pH.

#### 2.2.4 Ketolactose test

Most of the strains of *A. tumifaciens* and *A. radiobacter* have been found to produce 3-Ketolactose in lactose containing medium but not rhizobia. The composition of medium used for this test is same as that of the yeast mannitol agar except mannitol is replaced by lactose (10 g/l). The medium is poured in plates and on solidification the inoculum is streaked on it. After incubation when sufficient growth is observed, the media plates were flooded with Benedict's reagent. Development of yellow coloured ring of cuprous oxide (after 30 min to one hr) around the growth of organism is indicative of *Agrobacterium* contamination.

### 2.3 Nodulation tests

Nodulation tests were conducted by the following methods.

#### 2.3.1 Agar tube method

This method is good to study the nodulation and differentiation of symbiotic effectiveness with plants having small seeds. In this method the plants are wholly enclosed within the glass tube.

#### 2.3.2 Preparation of agar tubes

Sufficient SNA medium (1.0 g CaHPO_4_, 0.2 g K_2_HPO_4_, 0.2 g MgSO_4_•7H_2_O, 0.2 g NaCl, 0.1 g FeCl_3_, 8–15 g Agar, 1litre Distilled water, (1 ml/liter trace element solution) is added to the medium (15 ml for deep and 20 ml for slope) was put in the tubes (200 mm x 25 mm). The tubes were closed with cotton plugs with uniform depth (20 mm) and modest compactness. The tubes were autoclaved and set as agar deep tubes or slopes as required.

• **Nitrogen supplied control:** Nitrogen-controls were provided to a final concentration of approximately 70 ppm N (0.05% KNO_3_).
• **N-deficient control:** Agar tubes without inoculation are planted with seed or pre-germinated seedlings and put as uninoculated N-deficient control.

## 3 Experimental Design

### 3.1 Seed inoculation and plant growth assessment

Sterile soils in triplicates from six forest places were collected and brought to the laboratory. They were taken in pots (Triplicates) and the seeds inoculated with different rhizobial strain of respective species were sown and then the growth parameters of each plant were assessed as shown in Table 2.

**Table 2.** Screening and isolation of different indigenous rhizobial strains from host-specific *Vigna radiata* species from forest areas of Telangana.

		Nodulation			Plant Height (cm)		Plant dry weight (gm)		
Name of the plant	Rhizobial strain	No.	Size (mm)	Dry weight (mg)	Shoot	Root	Shoot	Root	N Content (%)
Uninoculated control	–	–	–	–	18.4 ±0.03	7.4 ±0.02	0.28 ±0.02	0.42 ±0.03	0.8 ±0.03

*Vigna radiata*	ET1	35	3	25±0.01	33.2 ±0.03	13.29 ±0.03	0.95 ±0.02	0.64 ±0.03	2.6 ±0.03
	JN1	19	1	16±0.01	29.3 ±0.02	12.8 ±0.03	0.88 ±0.02	0.57 ±0.02	2.4 ±0.03
	NP1	27	1	26±0.03	31.2 ±0.04	13.7 ±0.02	0.98 ±0.03	0.65 ±0.04	2.6 ±0.03
	BD1	40	3	33±0.05	38.9 ±0.04	18.4 ±0.03	1.02 ±0.03	0.68 ±0.02	2.9 ±0.01
	AB1	15	1	12±0.02	34.6 ±0.04	16.8 ±0.03	0.61 ±0.01	0.32 ±0.01	1.2 ±0.03
	KT1	35	3	27±0.03	35.6 ±0.03	15.2 ± 0.02	1.01 ±0.03	0.67 ±0.01	2.8 ±0.03

Undamaged and clean seeds of uniform size, selected to a reasonably uniform size were rinsed with 95% ethanol and immersed for 4 min in 0.2% HgCl_2_. Seeds were then washed five times with sterile water. The viable seeds are sown after sterilization and washing, either using two seeds per tube (seedlings later can be thinned to one) or singly, allowing sufficient extra tubes. After 8 weeks of growth, the growth parameters like nodulation efficiency, number of nodules, nodular dry weight, dry weight of the plant and the plant height were assessed.

**Fig. 1:**
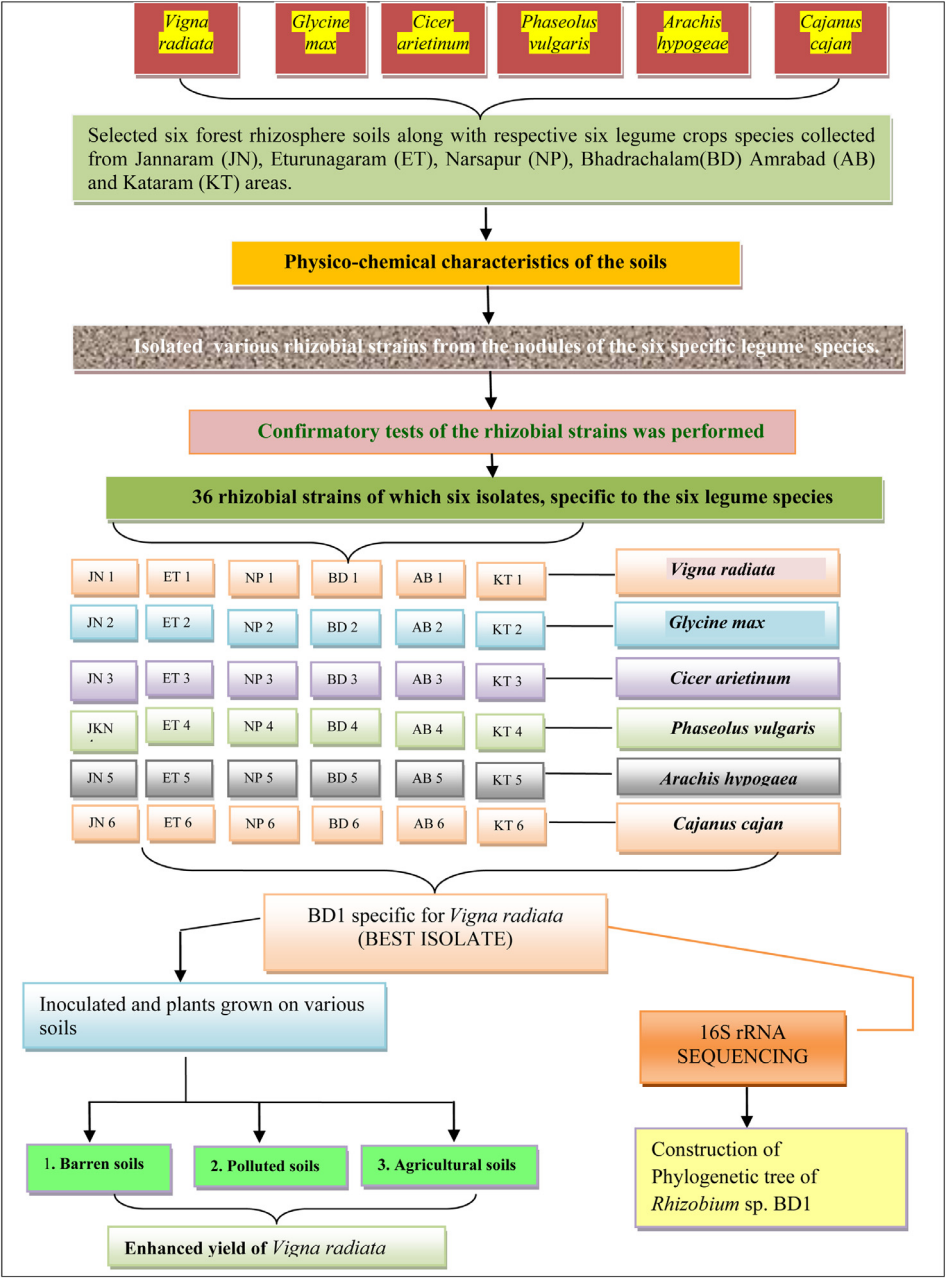
Steps involved in host specificity and effect of nodulation by indigenous rhizobial strain BD1 in *Vigna radiata*.

The presences of nodules, their nature at the time of harvesting that are pink because of leg-hemoglobin were accounted as effective. The rhizobial strains were isolated and tested for host specificity. All probable combination of the selected rhizobial isolates were checked under sterile conditions for improving nodulation to isolate the best host specificity plant as represented in Fig. 1.

### 3.2 Estimation of nitrogen in *Vigna radiata*

#### 3.2.1 Soil preparation and sowing

Soil is collected, vigorously washed and subjected to sterilization - to remove the nutrients and microorganisms particularly nitrogen fixing microorganisms from the soil to ensure that no external agent acts as source of nitrogen. The *Vigna radiata* seeds from a single batch and same size were sown in the pots filled with the soil (sterile and nutrient free) [5], [6], [7]. The pots were inoculated with specific bioinoculants (Nitrogen fixing Rhizobia under study) except the controls. Negative control is maintained without any nitrogen source except the nitrogen contained in the seed initially. Positive control is treated with sufficient/unlimited nitrogen source. All the pots are irrigated frequently with Nitrogen-free Hoagsland solution. After 60 days of sowing the plants are extricated for examination of parameters.

#### 3.2.2 Estimation of nitrogen by [4] method

Estimation of nitrogen content in plant part is the best way to analyze the efficiency of Nitrogen fixation by legume-Rizobium symbiosis [8]. This is done by the Snell and Snell method. Dried and well ground plant sample (0.1 g) is completely digested with H_2_SO_4_ and heat it. Then again add H_2_O_2_ till it becomes colourless. Then the intensity of color developed upon treating with NaOH, sodium silicate and Nesseler's reagent is measured at 440 nm (blue filter) in the colorimeter. The OD values obtained are plotted on the standard graph. The Nitrogen content is measured from the standard graph.

**Table 3:** Effects of Nodulation in *Vigna radiata* by BD-1isolate in agricultural, barren and polluted soils.

		Nodulation			Plant Height(cm)		Plant dry weight (gm)		
Name of the plant	Soil types	No.	Size (µm)	Dry weight (mg)	Shoot	Root	Shoot	Root	N content (%)
**Control**		–	–	–	18.30 ±0.05	13.20 ± 0.03	4.97 ±0.03	0.463 ±0.04	0.90 ±0.03

***Vigna radiata* sp.**	**Agricultural soils**	32 ±0.05	330 ±0.02	250 ± 0.01	38.56 ±0.05	37.9 ± 0.03	14.07 ±0.04	1.68 ±0.05	2.76 ±0.02
	**Polluted soils**	29± 0.04	140 ±0.01	1080 ± 0.05	35.7 ± 0.05	39.13 ± 0.02	12.12 ±0.03	1.82 ±0.04	1.63 ±0.03

**Barren soils**	33± 0.05	350 ±0.01	190 ± 0.01	33.06 ±0.03	37.06 ±0.03	11.94 ±0.02	1.63 ±0.04	2.50 ±0.02

#### 3.2.3 Collection of soil samples from different problematical sites

Soil samples from different sites such as polluted soils, barren soils and agriculture soils were taken in triplets and inoculated with rhizobial strains in order to check the improvement in nodulation and development of these respective plants during inoculation in field trials (Table 3).

## Ethics Statement

This study doesn't use experiment on human or an animal.

## CRediT authorship contribution statement

**Sanjeev Kumar K:** Formal analysis, Writing – review & editing. **Pavan Kumar Pindi:** Visualization, Writing – original draft, Writing – review & editing.

## Declaration of Competing Interest

The authors declare that they have no known competing financial interests or personal relationships, which have or could be perceived to have influenced the work reported in this article.

## Appendix Supplementary materials

Image, application 1
