# Supplementary material for: Data on host specificity and symbiotic association between indigenous Rhizobium BD1 strain and Vigna radiata (green gram)
Source: Data Brief. 2021 Oct 27;39:107520. doi: 10.1016/j.dib.2021.107520 (PMC8581496; doi:10.1016/j.dib.2021.107520)
Supplement: Supplementary file 1 [file mmc1.docx]

**Table 2:** Screening and isolation of different indigenous rhizobial strains from host-specific *Vigna radiata* species from forest areas of Telangana.

| Name of the plant | Rhizobial strain | Nodulation | | | Plant Height(cm) | | Plant dry weight (gm) | | N Content (%) |
| --- | --- | --- | --- | --- | --- | --- | --- | --- | --- |
|  |  | No. | Size(mm) | Dry weight  (mg) | Shoot | Root | Shoot | Root |  |
| Uninoculated control | - | - | - | - | 18.4  ±0.03 | 7.4  ±0.02 | 0.28  ±0.02 | 0.42  ±0.03 | 0.8  ±0.03 |
| *Vigna radiata* | ET1 | 35 | 3 | 25±0.01 | 33.2  ±0.03 | 13.29  ±0.03 | 0.95  ±0.02 | 0.64  ±0.03 | 2.6  ±0.03 |
|  | JN1 | 19 | 1 | 16±0.01 | 29.3  ±0.02 | 12.8  ±0.03 | 0.88  ±0.02 | 0.57  ±0.02 | 2.4  ±0.03 |
|  | NP1 | 27 | 1 | 26±0.03 | 31.2  ±0.04 | 13.7  ±0.02 | 0.98  ±0.03 | 0.65  ±0.04 | 2.6  ±0.03 |
|  | BD1 | 40 | 3 | 33±0.05 | 38.9  ±0.04 | 18.4  ±0.03 | 1.02  ±0.03 | 0.68  ±0.02 | 2.9  ±0.01 |
|  | AB1 | 15 | 1 | 12±0.02 | 34.6  ±0.04 | 16.8  ±0.03 | 0.61  ±0.01 | 0.32  ±0.01 | 1.2  ±0.03 |
|  | KT1 | 35 | 3 | 27±0.03 | 35.6  ±0.03 | 15.2  0.02 | 1.01  ±0.03 | 0.67  ±0.01 | 2.8  ±0.03 |

**Raw data for above table:**

Table: 1 Nitrogen Content

| S.No | Rhizobial Strain | S1 | S2 | S3 | Mean | Std. Dev. |
| --- | --- | --- | --- | --- | --- | --- |
| 1 | Control | 0.8 | 0.77 | 0.83 | 0.8 | 0.03 |
| 2 | ET1 | 2.59 | 2.6 | 2.65 | 2.6 | 0.0321 |
| 3 | JN1 | 2.4 | 2.35 | 2.41 | 2.38 | 0.032 |
| 4 | NP1 | 2.68 | 2.61 | 2.65 | 2.64 | 0.035 |
| 5 | BD1 | 2.88 | 2.9 | 2.9 | 2.89 | 0.011 |
| 6 | AB1 | 1.21 | 1.15 | 1.2 | 1.18 | 0.0321 |
| 7 | KT1 | 2.81 | 2.83 | 2.77 | 2.8 | 0.03 |

Table: Nodulation Parameters: Number, size and Dry weight

| S.No | Rhizobial Strain | Nodule number | | | |
| --- | --- | --- | --- | --- | --- |
|  |  | S1 | S2 | S3 | Mean |
| 1 | Control | - | - | - | - |
| 2 | ET1 | 34 | 38 | 33 | 35 |
| 3 | JN1 | 17 | 20 | 20 | 19 |
| 4 | NP1 | 29 | 27 | 25 | 27 |
| 5 | BD1 | 42 | 43 | 36 | 40 |
| 6 | AB1 | 13 | 15 | 18 | 15 |
| 7 | KT1 | 34 | 38 | 33 | 35 |

| S.No | Rhizobial Strain | Nodule size | | | |
| --- | --- | --- | --- | --- | --- |
|  |  | S1 | S2 | S3 | Mean |
| 1 | Control | - | - | - | - |
| 2 | ET1 | 3 | 3 | 3 | 3 |
| 3 | JN1 | 1 | 1 | 1 | 1 |
| 4 | NP1 | 1 | 1 | 1 | 1 |
| 5 | BD1 | 3 | 3 | 3 | 3 |
| 6 | AB1 | 1 | 1 | 1 | 1 |
| 7 | KT1 | 3 | 3 | 3 | 3 |

| S.No | Rhizobial Strain | Nodule dry weight | | | | |
| --- | --- | --- | --- | --- | --- | --- |
|  |  | S1 | S2 | S3 | Mean | Std. Dev. |
| 1 | Control |  |  |  |  |  |
| 2 | ET1 | 25.00 | 25.01 | 24.99 | 25 | 0.01 |
| 3 | JN1 | 15.99 | 16.00 | 16.01 | 16 | 0.01 |
| 4 | NP1 | 26.00 | 26.01 | 25.95 | 25.98 | 0.032 |
| 5 | BD1 | 33.05 | 33.12 | 33.15 | 33.10 | 0.051 |
| 6 | AB1 | 12.05 | 12 | 12.02 | 12.023 | 0.025 |
| 7 | KT1 | 27.00 | 27.05 | 27.06 | 27.03 | 0.032 |

Table: Plant height

| S.No | Rhizobial Strain | Plant Shoot height | | | | | Plant Root height | | | | |
| --- | --- | --- | --- | --- | --- | --- | --- | --- | --- | --- | --- |
|  |  | S1 | S2 | S3 | Mean | Std. Dev | S1 | S2 | S3 | Mean | Std. Dev. |
| 1 | Control | 18.45 | 18.49 | 18.43 | 18.45 | 0.03 | 7.40 | 7.36 | 7.4 | 7.4 | 0.023 |
| 2 | ET1 | 33.15 | 33.2 | 33.2 | 33.18 | 0.028 | 13.32 | 13.29 | 13.25 | 13.29 | 0.035 |
| 3 | JN1 | 29.30 | 29.28 | 29.32 | 29.3 | 0.02 | 12.8 | 12.83 | 12.86 | 12.83 | 0.03 |
| 4 | NP1 | 31.2 | 31.25 | 31.17 | 31.2 | 0.04 | 13.65 | 13.7 | 13.71 | 13.68 | 0.032 |
| 5 | BD1 | 38.89 | 38.95 | 38.87 | 38.9 | 0.041 | 18.35 | 18.4 | 18.4 | 18.38 | 0.028 |
| 6 | AB1 | 34.57 | 34.65 | 34.6 | 34.6 | 0.04 | 16.81 | 16.82 | 16.76 | 16.79 | 0.032 |
| 7 | KT1 | 35.6 | 35.6 | 35.65 | 35.61 | 0.028 | 15.21 | 15.18 | 15.22 | 15.20 | 0.02 |

Table: plant dry weight

| S.No | Rhizobial Strain | Plant shoot dry weight | | | | | Plant Root dry weight | | | | |
| --- | --- | --- | --- | --- | --- | --- | --- | --- | --- | --- | --- |
|  |  | S1 | S2 | S3 | Mean | Std. Dev | S1 | S2 | S3 | Mean | Std.Dev. |
| 1 | Control | 0.28 | 0.3 | 0.26 | 0.28 | 0.02 | 0.42 | 0.45 | 0.39 | 0.42 | 0.03 |
| 2 | ET1 | 0.95 | 0.98 | 0.94 | 0.95 | 0.02 | 0.64 | 0.67 | 0.6 | 0.64 | 0.035 |
| 3 | JN1 | 0.85 | 0.88 | 0.88 | 0.88 | 0.017 | 0.55 | 0.59 | 0.57 | 0.57 | 0.02 |
| 4 | NP1 | 0.98 | 1.02 | 0.96 | 0.98 | 0.03 | 0.65 | 0.7 | 0.62 | 0.65 | 0.04 |
| 5 | BD1 | 1.02 | 1.05 | 0.98 | 1.016 | 0.035 | 0.68 | 0.7 | 0.66 | 0.68 | 0.02 |
| 6 | AB1 | 0.59 | 0.62 | 0.61 | 0.61 | 0.015 | 0.31 | 0.32 | 0.33 | 0.32 | 0.01 |
| 7 | KT1 | 1.01 | 1.05 | 0.98 | 1.01 | 0.035 | 0.66 | 0.68 | 0.67 | 0.67 | 0.01 |

**Table 3:** Effects of Nodulation in *Vigna radiata* by BD-1isolate in agricultural, barren and polluted soils

| **Name of the plant** | **Soil types** | | **Nodulation** | | | **Plant Height(cm)** | | **Plant dry weight (gm)** | | **N content (%)** |
| --- | --- | --- | --- | --- | --- | --- | --- | --- | --- | --- |
|  |  |  | **No.** | Size  (µm) | **Dry weight**  **(mg)** | **Shoot** | **Root** | **Shoot** | **Root** |  |
| **Control** | | | - | - | - | 18.30  ±0.05 | 13.20  ± 0.03 | 4.97  ±0.03 | 0.463  ±0.04 | 0.90  ±0.03 |
| ***Vigna radiata* sp.** | | **Agricultural soils** | 32 | 330 | 250  ± 0.01 | 38.56  ±0.05 | 37.9  ± 0.03 | 14.07  ±0.04 | 1.68  ±0.05 | 2.76  ±0.02 |
|  |  | **Polluted soils** | 29 | 140 | 1080 ±0.05 | 35.7  ± 0.05 | 39.13  ± 0.02 | 12.12  ±0.03 | 1.82  ±0.04 | 1.63  ±0.03 |
|  |  | **Barren soils** | 33 | 350 | 190  ± 0.01 | 33.06  ±0.03 | 37.06  ±0.03 | 11.94  ±0.02 | 1.63  ±0.04 | 2.50  ±0.02 |

**Raw data for above table**

Table: 1 Nitrogen Content

| S.No | Soil type | S1 | S2 | S3 | Mean | Std. Dev. |
| --- | --- | --- | --- | --- | --- | --- |
| 1 | Control | 0.90 | 0.87 | 0.93 | 0.90 | 0.03 |
| 2 | Agricultural soils | 2.79 | 2.76 | 2.75 | 2.76 | 0.02 |
| 3 | Polluted soils | 1.63 | 1.66 | 1.59 | 1.626 | 0.035 |
| 4 | Barren soils | 2.50 | 2.52 | 2.48 | 2.50 | 0.02 |

Table: plant dry weight

| S. No | Soil type | Plant shoot dry weight | | | | | Plant Root dry weight | | | | |
| --- | --- | --- | --- | --- | --- | --- | --- | --- | --- | --- | --- |
|  |  | S1 | S2 | S3 | Mean | Std. Dev | S1 | S2 | S3 | Mean | Std. Dev. |
| 1 | Control | 5 | 4.97 | 4.94 | 4.97 | 0.03 | 0.463 | 0.5 | 0.426 | 0.463 | 0.037 |
| 2 | Agricultural soils | 14.07 | 14.11 | 14.03 | 14.07 | 0.04 | 1.68 | 1.74 | 1.64 | 1.68 | 0.05 |
| 3 | Polluted soils | 12.15 | 12.12 | 12.08 | 12.12 | 0.035 | 1.78 | 1.82 | 1.86 | 1.82 | 0.04 |
| 4 | Barren soils | 11.94 | 11.92 | 11.96 | 11.94 | 0.02 | 1.63 | 1.58 | 1.67 | 1.63 | 0.04 |

| S.No | Rhizobial Strain | Plant Shoot height | | | | | Plant Root height | | | | |
| --- | --- | --- | --- | --- | --- | --- | --- | --- | --- | --- | --- |
|  |  | S1 | S2 | S3 | Mean | Std. Dev | S1 | S2 | S3 | Mean | Std. Dev. |
| 1 | Control | 18.3 | 18.35 | 18.25 | 18.3 | 0.05 | 13.2 | 13.23 | 13.17 | 13.2 | 0.03 |
| 2 | Agricultural soils | 38.56 | 38.61 | 38.51 | 38.56 | 0.05 | 37.87 | 37.9 | 37.93 | 37.9 | 0.03 |
| 3 | Polluted soils | 35.7 | 35.75 | 35.65 | 35.7 | 0.05 | 39.13 | 39.11 | 39.15 | 39.13 | 0.02 |
| 4 | Barren soils | 33.03 | 33.06 | 33.09 | 33.06 | 0.03 | 37.06 | 37.03 | 37.09 | 37.06 | 0.03 |

Table: Nodulation Parameters: Number, size and Dry weight

| S. No | Rhizobial Strain | Nodule number | | | |
| --- | --- | --- | --- | --- | --- |
|  |  | S1 | S2 | S3 | Mean |
| 1 | Control | - | - | - | - |
| 2 | Agricultural soils | 35 | 30 | 31 | 32 |
| 3 | Polluted soils | 30 | 25 | 32 | 29 |
| 4 | Barren soils | 35 | 33 | 31 | 33 |

| S. No | Rhizobial Strain | Nodule size (µm) | | | |
| --- | --- | --- | --- | --- | --- |
|  |  | S1 | S2 | S3 | Mean |
| 1 | Control | - | - | - | - |
| 2 | Agricultural soils | 330 | 330 | 330 | 330 |
| 3 | Polluted soils | 140 | 140 | 140 | 140 |
| 4 | Barren soils | 350 | 350 | 350 | 350 |

| S. No | Rhizobial Strain | Nodule dry weight | | | | |
| --- | --- | --- | --- | --- | --- | --- |
|  |  | S1 | S2 | S3 | Mean | Std. Dev. |
| 1 | Control | - | - | - | - | - |
| 2 | Agricultural soils | 25.0 | 25.01 | 25.02 | 25.01 | 0.01 |
| 3 | Polluted soils | 10.8 | 10.75 | 10.85 | 10.8 | 0.05 |
| 4 | Barren soils | 19.01 | 19 | 19.02 | 19.01 | 0.01 |
